# Supplementary material for: Hybrid Models and Biological Model Reduction with PyDSTool
Source: PLoS Comput Biol. 2012 Aug 9;8(8):e1002628. doi: 10.1371/journal.pcbi.1002628 (PMC3415397; doi:10.1371/journal.pcbi.1002628)
Supplement: Text S4 — Complete source code for the PyDSTool package (version 0.88.120504). Includes API documentation and help files linking to web pages. This file is identical to the current public release on Sourceforge.net. (ZIP) [file pcbi.1002628.s004.zip › PyDSTool/html/PyDSTool.ModelConstructor'.GDescriptor-class.html]

xml version="1.0" encoding="ascii"?


PyDSTool.ModelConstructor'.GDescriptor


| Home | Trees | Indices | Help | PyDSTool |
| --- | --- | --- | --- | --- |

Package PyDSTool ::
Module ModelConstructor' ::
Class GDescriptor

[hide private]

[frames] | no frames]

# Class GDescriptor

source code

```
 object --+        
          |        
common.args --+    
              |    
     Descriptor --+
                  |
                 GDescriptor
```

---

All-in-one descriptor class for single Generators, and information
necessary to be able to build a Model object using a ModelConstructor
call -- i.e. for forming a valid 'generatorspecs' field.


|  |  |
| --- | --- |
| Instance Methods | [hide private] |

|  |  |
| --- | --- |
| \_\_getitem\_\_(self, hier\_name)  Return object in model spec named using the hierarchical naming format. | source code |

|  |  |
| --- | --- |
| isinstantiable(self) | source code |

|  |  |
| --- | --- |
| search(self, hier\_name) | source code |

|  |  |
| --- | --- |
| validate(self) | source code |

**Inherited from `Descriptor`**:
`__init__`,
`__repr__`,
`__str__`

**Inherited from `common.args`**:
`__add__`,
`__cmp__`,
`__contains__`,
`__delitem__`,
`__eq__`,
`__ge__`,
`__gt__`,
`__iter__`,
`__le__`,
`__len__`,
`__lt__`,
`__ne__`,
`__setitem__`,
`clear`,
`copy`,
`fromkeys`,
`get`,
`has_key`,
`info`,
`items`,
`iteritems`,
`iterkeys`,
`itervalues`,
`keys`,
`pop`,
`popitem`,
`setdefault`,
`update`,
`values`

**Inherited from `common.args`** (private):
`_infostr`

**Inherited from `object`**:
`__delattr__`,
`__getattribute__`,
`__hash__`,
`__new__`,
`__reduce__`,
`__reduce_ex__`,
`__setattr__`


|  |  |
| --- | --- |
| Class Variables | [hide private] |

\_checkKeys = `'target'`

\_defaults = `{'description': '', 'options': {}, 'unravelInfo': ...`

\_validKeys = `('changelog', 'orig_name', 'modelspec', 'descript...`


|  |  |
| --- | --- |
| Properties | [hide private] |

**Inherited from `object`**:
`__class__`


|  |  |
| --- | --- |
| Method Details | [hide private] |

|  |  |
| --- | --- |
| \_\_getitem\_\_(self, hier\_name)  *(Indexing operator)* | source code |

Return object in model spec named using the hierarchical naming
format.

Overrides: common.args.\_\_getitem\_\_

|  |  |
| --- | --- |
| validate(self) | source code |

Overrides: Descriptor.validate

  


|  |  |
| --- | --- |
| Class Variable Details | [hide private] |

### \_defaults

Value:
:   ```
    {'description': '',
     'options': {},
     'unravelInfo': True,
     'withJac': False,
     'withJacP': False,
     'withStdEvts': False}
    ```

### \_validKeys

Value:
:   ```
    ('changelog',
     'orig_name',
     'modelspec',
     'description',
     'algparams',
     'target',
     'withStdEvts',
     'stdEvtArgs',
    ...
    ```

  


| Home | Trees | Indices | Help | PyDSTool |
| --- | --- | --- | --- | --- |

|  |  |
| --- | --- |
| Generated by Epydoc 3.0.1 on Fri May 4 15:24:07 2012 | http://epydoc.sourceforge.net |
